# Self-Identified Employment Challenges for Young Adults with a Cleft Lip and Palate: A Qualitative Exploration

**DOI:** 10.3390/bs15010091

**Published:** 2025-01-20

**Authors:** Haslina Rani, Hasherah Mohd Ibrahim, Nurshazwanie Amira Mohamed Noor Shafie, Suziyani Mohamed, Azianura Hani Shaari, Murshida Marizan Nor, Tuti Ningseh Mohd-Dom

**Affiliations:** 1 Faculty of Dentistry, Universiti Kebangsaan Malaysia, Kuala Lumpur 50300, Malaysia; hr@ukm.edu.my (H.R.); murshida@ukm.edu.my (M.M.N.); 2 Family Oral Wellness Research Group, Universiti Kebangsaan Malaysia, Kuala Lumpur 50300, Malaysia; 3 Faculty of Health Sciences, Universiti Kebangsaan Malaysia, Kuala Lumpur 50300, Malaysia; hasherah@ukm.edu.my (H.M.I.); nsamirashafie@gmail.com (N.A.M.N.S.); 4 Faculty of Education, Universiti Kebangsaan Malaysia, Bangi 43600, Malaysia; suziyani@ukm.edu.my; 5 Faculty of Social Sciences and Humanities, Universiti Kebangsaan Malaysia, Bangi 43600, Malaysia; azianura@ukm.edu.my

**Keywords:** cleft lip and palate, psychology, behavioural sciences, occupation, self-esteem, multidisciplinary

## Abstract

As individuals with a cleft lip and palate (CLP) transition into adulthood, they face unique employment challenges related to income, job stability, and fewer career options. This study explored these challenges through two focus group discussions with 19 participants (aged 21–38), primarily women, to understand their employment experiences. Thematic analysis revealed the following three main themes: (1) physical factors, (2) psychosocial factors, and (3) overcoming employment challenges, with nine sub-themes including speech, hearing, appearance, health, childhood experiences, societal expectations, lack of self-confidence, communication improvement, and self-esteem building. The findings highlighted that physical and psychosocial factors significantly shape employment outcomes for CLP individuals. Difficulties with speech often hinder professional communication, while appearance concerns can reduce confidence in interviews and workplaces. To address these issues, the participants used strategies like targeted speech therapy and self-esteem building, which helped improve their communication and resilience. This study emphasises the need for targeted interventions such as specialised career counselling, access to assistive technologies, and inclusive workplace policies to support CLP individuals in overcoming employment barriers and achieving stable careers.

## 1. Introduction

Cleft lip and palate (CLP) is one of the most common craniofacial birth defects globally, affecting approximately 10.8 million people as of 2017, with the highest prevalence in low- and middle-income countries (Chaudhary, 2022). While its exact aetiology remains unresolved, CLP results from fusion failure of the mouth’s structural components during the first trimester of pregnancy (Tahmeedullah & Hayat, 2022). The condition manifests in visible facial differences and functional impairments, contributing to stigma and bullying from an early age, which often persist into adulthood and impact self-esteem, social integration, and communication abilities (Nicholls et al., 2018; Sahoo et al., 2023).

In regions with resources for multidisciplinary care, individuals with a CLP benefit from tailored treatments at various life stages (Choi et al., 2021; Felton et al., 2018), although certain therapies lack consistent evidence for their effectiveness (Ma et al., 2021). However, in areas with limited awareness and treatment access, many individuals with a CLP face lifelong barriers due to suboptimal medical and social support (Alonso et al., 2013). In Malaysia, while a birth defect registry is unavailable, studies suggest that about 1 in 591 live births is affected (Thong et al., 2005). Combined cleft clinics in some hospitals follow management protocols that typically end by age twenty, leaving gaps in support as individuals transition to adulthood and seek stable employment (Hussin et al., 2022).

While individuals with a CLP around the world encounter various physical and psychological challenges that hinder their social integration and professional advancement, in countries like Malaysia, these are compounded by cleft-specific cultural stigmas and limited public awareness, which may contribute to employment discrimination (Hasanuddin et al., 2023; Hussin et al., 2022). In contrast to high-income nations, Malaysia’s healthcare resources are often unevenly distributed, and employment policies lack provisions for individuals with visible differences, complicating their access to meaningful employment opportunities.

This study employs a phenomenological, qualitative approach, using focus groups to deeply explore the unique employment challenges faced by young adults with a CLP. This approach allows for a rich examination of the personal and social dimensions that quantitative methods might overlook, such as the emotional toll of social stigma and its impact on career choices. Focus groups create a supportive setting for participants to share their lived experiences, providing insights into both internalised and external employment barriers. By capturing individuals’ narratives, this study aims to identify themes specific to the employment challenges faced by CLP individuals in Malaysia, offering a contextually grounded understanding of their experiences.

### Literature Review

Young adults with a CLP commonly face a multitude of physical and psychological challenges. Physically, they often experience issues related to nasal obstruction, sleep disturbances, and mandibular function impairment, with a significant subset reporting moderate to severe dysfunction in these areas (S. E. Braun et al., 2023). Speech difficulties are also prevalent, as evidenced by lower scores on the CLEFT-Q Speech modules among many patients (Mcwilliams et al., 2023; Nicholls et al., 2019a). Additionally, anatomical variations can lead to complications in hearing, feeding, and social integration, necessitating a multidisciplinary approach involving orthodontists, speech therapists, and psychologists (Gandhi et al., 2020; Muzammil et al., 2021).

Psychologically, individuals with a CLP are at risk of developing facial dysmorphic disorder, a variant of body dysmorphic disorder, due to their preoccupation with perceived facial defects (Stepp et al., 2023). This can lead to significant psychological burdens, including a negative body image and social anxiety, which are exacerbated by the unique psychosocial challenges encountered at various developmental stages (Mcwilliams et al., 2023). The long-term treatment pathway, often extending into adulthood, requires the careful management of transitions in care to ensure holistic, patient-centred support (Nicholls et al., 2019a).

As they transition into adulthood, individuals with a CLP, like their peers, confront critical life milestones, including career and job prospects. Yet, unlike others, they encounter distinct challenges in employment that are significant and unique to their condition. One primary issue is the stigma and social exclusion they experience from a young age, which can lead to a reduced self-esteem and social skills, impacting their professional interactions and opportunities later in life (Arias-Urueña et al., 2023). Additionally, the need for ongoing interdisciplinary treatment, including surgeries and therapies, often results in significant time away from work or school, further hindering career progression and stability (S. E. Braun et al., 2023; Cerón-Zapata et al., 2023). Financial barriers and a lack of awareness about available clinical options also pose significant challenges, as many adults with a CLP continue to face unresolved functional issues such as speech and nasal breathing difficulties, which can affect their job performance and employability (Paradowska-Stolarz et al., 2022). The necessity of specialised orthodontic and surgical interventions, which are often costly and time-consuming, adds another layer of difficulty in maintaining consistent employment (Garain et al., 2022). Moreover, the psychological impact of bullying and social isolation, frequently discussed on platforms like Twitter, highlights the ongoing emotional and mental health struggles that can affect workplace dynamics and career advancement (Marya et al., 2022).

Societal perceptions significantly impact employment opportunities for individuals with a cleft lip and palate. Research highlights that the social stigma surrounding cleft lip and palate can lead to disadvantages in various aspects, including employment (Chung et al., 2019). Moreover, societal beliefs attributing cleft lip and palate to supernatural forces or contagions can result in individuals with these conditions being devalued, sometimes to the extent of not being considered human (Chattopadhyay et al., 2020; Lotfallah et al., 2023). This negative perception can create barriers to securing employment opportunities, as individuals with a cleft lip and palate may face discrimination or prejudice in the workplace due to misconceptions and biases held by society.

While general employment challenges affect all individuals, those with a CLP face a complex set of hurdles spanning medical, psychological, and social aspects, highlighting the need for comprehensive support to enhance their employability. Despite Malaysia’s commitment to social inclusivity, the specific challenges faced by and contributions of CLP individuals to national development are often overlooked. This study explores the employment barriers faced by CLP individuals in Malaysia, with the goal of advancing their inclusion and maximising their potential.

## 2. Materials and Methods

### 2.1. Research Design

A qualitative methodology was selected to deeply investigate the perspectives and experiences of young adults regarding job-seeking and employment opportunities, specifically examining if having a CLP poses a barrier. This study used the phenomenology approach, which is a type of qualitative research that is used to explore multiple perspectives and deeply understand the lived experiences of individuals. Two focus group discussions (FGDs) were conducted, with semi-structured questions covering the aforementioned aspects. Facilitators with training and experience led the discussions to ensure the standardisation and reliability of questioning methods. And, while preparing this article, the authors used ChatGPT v.4 to improve on language and readability; used SciSpace v.1to search for relevant literature, and used Mendeley v.2 for citation references.

### 2.2. Participants

Participants were chosen from two clinics in UKM and provided by the Cleft Lip and Palate Association of Malaysia (CLAPAM). Eligible Malaysians aged 15–40 were contacted using the telephone number recorded at the orthodontics clinic and CLAPAM office. Those who were not contactable were excluded from the study. Brief explanations of the research were given and those who agreed to participate were given further details on the time and venue of the FGD. In total, 56 individuals were contacted, and 35 individuals were not interested in participating. In total, 21 agreed to attend the FGD, but only 19 turned up on the set date, citing unavoidable circumstances.

Table 1 summarises the background of the FGD participants, who were aged between 21 and 38 years. The group included 17 women and 2 men, with most participants having attained post-secondary education. Many were either salaried employees or self-employed, while one participant (P5) was still pursuing her studies, and another (P10), a physiotherapy graduate, was seeking employment. Most participants reported earning a monthly income from MYR 1000 to MYR 3000. Their occupations and years of experience in their current roles varied widely, reflecting a diverse range of employment experiences.

### 2.3. Data Collection

Prior to the FGD, the lead researcher (TNMD) gave an overview of the procedure to all participants, and reiterated the risks and benefits of the study, as well as the ground rules of speaking their views and listening to others. Confidentiality in reports was reassured, and written consent was obtained from the participants before the FGD started. The participants were divided into two groups, and each FGD was led by an experienced moderator (TNMD and AHS). Two scribes assisted the facilitators in recording the sessions, which were held in rooms equipped with audio-visual systems. Data were collected through audio and video recordings for analysis purposes. During the discussion, the facilitators probed, paused, and reflected appropriately and observed signs of non-verbal cues among participants. Each session was about 60 min in length each, and at the end of the session, the facilitators thanked the participants for their contributions to the discussion.

### 2.4. Data Analysis

All transcripts of the audio data from the FGDs were transcribed verbatim. Referral to the videos was made whenever there was a need to clarify the pronunciation of words, the manner in which they were spoken, or the identity of the speaker. A thematic analysis was then undertaken following the guidelines for thematic analysis specified by Braun and Clarke (V. Braun & Clarke, 2006) First, the initial analysts, NA and AN, read and reread the transcripts to familiarise themselves with the dataset. Both were also present and scribed notes during the FGD sessions. Second, both identified initial codes and developed an initial coding frame. To enhance data triangulation, these codes were presented to six research team members (NAMNS, HMI, AHS, TNMD, MMN, and HR), and any discrepancies in codes were discussed until consensus was achieved. Some of the team members are clinicians who treat cleft patients and are familiar with issues such as speech and self-esteem. Third, once all the transcripts had been coded, the team members explored possible emerging themes that would echo the meanings of the participants’ reflections. Fourth, the emerging themes were reviewed, and once again, discrepancies were discussed until consensus was achieved.

ZA and ZG are members of a cleft support NGO who were present at the FGDs and reviewed the final themes and sub-themes. Data remained in the Malay language during the analysis and illustrative quotes were eventually translated into English by researchers proficient in both languages (HR and TNMD).

### 2.5. Reflexivity

The research team included clinicians who are involved in providing cleft care and members of a cleft support NGO, whose combined expertise enriched the study. The clinicians provided insights into physical and healthcare-related challenges, while the NGO members highlighted psychosocial aspects based on lived experiences. These perspectives offered a holistic understanding of the challenges faced by the participants, but also posed the risk of bias. To mitigate this, the FGDs and initial thematic analysis were conducted by team members without direct involvement in cleft care, ensuring that the participants’ narratives were interpreted without preconceived notions. Regular team discussions were held to reflect on how our experiences shaped the research process, helping to balance professional insights with an open, participant-centred approach. 

#### Ethical Considerations

This study adhered to the Declaration of Helsinki and received approval from the Universiti Kebangsaan Malaysia Research and Ethics Committee (Reference: UKM PPI/111/8/JEP-2019-632). Written informed consent was obtained, and the participants were informed of their voluntary participation, right to withdraw, and data confidentiality. The FGDs were conducted in a secure, private setting with only the research team and participants present. Sensitive topics were handled carefully, allowing the participants to skip questions or pause discussions if needed. Emotional support resources were provided through a cleft support NGO, ensuring a safe and supportive environment for the participants to share their experiences.

## 3. Results

This study identified three major themes and nine sub-themes regarding employment opportunities through thematic analysis (Table 2). The major themes were physical factors, psychosocial factors, and overcoming challenges. The physical factors included speech, hearing, appearance, and health issues. The psychosocial factors included childhood experiences, societal expectations, and lack of self-esteem. Overcoming challenges included improving communication skills and building self-esteem.

### 3.1. Theme 1: Physical Factors

The research found that physical traits play a crucial role in the employability of people with a CLP. It further explored how speech, hearing, and appearance impact job opportunities based on participants’ experiences. The study also recognised worries about the potential impacts of health issues on daily job tasks.

#### 3.1.1. Sub-Theme 1: Speech Intelligibility

The participants highlighted speech challenges as a significant barrier to effective verbal communication, particularly in fields that require clear interaction, such as healthcare. Issues like a hypernasal pitch, unclear pronunciation, and difficulties being understood often affected their self-confidence and limited their career opportunities.

P3 succinctly explained the core issue, as follows: “I cannot speak clearly. People find it hard to understand me”. For individuals with a cleft lip and palate (CLP), these challenges often create a disconnect between their abilities and how they are perceived by others, particularly employers. P14 shared her struggles in pursuing a nursing career, where effective communication is crucial for patient care. “When you have nasal sound [when you speak], [it is] difficult for people to understand. The way we speak is the reason people don’t want to employ you. Because maybe they are afraid people won’t understand us later [if we get the job]”. Despite attending multiple job interviews, she was unable to secure a nursing position, as employers expressed concerns about her ability to interact with patients effectively, particularly in explaining medical procedures or medications. These experiences left her feeling that her speech challenges directly influenced her employability in communication-dependent roles.

For P19, her nasal voice and difficulty pronouncing words created anxiety about how others perceived her. “So, when I want to speak, I am worried people won’t understand me. And if it’s in at the workplace, I’ll feel more worried, maybe I feel people think I am not capable”. This fear of misunderstanding not only affected her interactions, but also reinforced feelings of inadequacy, further lowering her confidence in professional and social settings. Similarly, P10 shared how his speech challenges impacted his practical training as a physiotherapy student, a role that requires consistent communication with patients. “I had [to do] practical [work] when I was studying (physiotherapy). I need to communicate with patients. So sometimes when patients do not understand [what I’m saying], it makes my self-confidence low”. Misunderstandings during patient interactions left him questioning his ability to succeed in his chosen field, despite his qualifications and efforts. P10 was still seeking employment during this period.

#### 3.1.2. Sub-Theme 2: Hearing Impairment

Effective communication relies not only on clear speech, but also on good listening skills, which can be significantly hindered by hearing impairments. Two participants in this study shared how their hearing challenges have impacted their interactions with colleagues and clients, often complicating workplace communication.

P9 described how her hearing difficulties necessitated the use of adaptive strategies to understand others. “I don’t hear well. I have hearing problems. I sometimes need to use hearing aid or I lip-read. Otherwise, hard for me to know what people say”. Her reliance on hearing aids and lip-reading highlights the additional effort required for her to navigate conversations, particularly in work settings where clear communication is crucial.

For P13, hearing challenges began in early childhood, profoundly influencing her speech development and social interactions. “I learned to speak late because I had hearing problems. My teacher thought I was autistic. I was 10 years old when my teacher discovered I could speak. I did not learn to speak because I did not hear well, and no one really noticed this when I was young. My mother said, when I was small, most of the time, I just kept quiet. Now I use hearing aid”. Her delayed speech development, initially misunderstood as autism, underscores the challenges in identifying and addressing hearing issues in children, particularly when they are not immediately apparent. The absence of early intervention limited her ability to communicate effectively during her formative years, which had lasting effects on her confidence and ability to engage in conversations.

Both participants’ experiences highlight the compounding challenges of hearing impairments for individuals with a cleft lip and palate (CLP), as these difficulties can exacerbate existing barriers related to speech and self-esteem. Misunderstandings in communication not only hinder the professional effectiveness of these individuals, but also impact their relationships with colleagues and clients.

#### 3.1.3. Sub-Theme 3: Perceptions of Appearance

The participants in this study largely expressed that their cleft condition did not overtly limit their employment opportunities or lead to outright discrimination. Many believed that factors such as confidence, skills, and qualifications were more significant in securing a job than physical appearance. However, some shared critical perspectives on how societal expectations around appearance could subtly influence job-seeking processes or the types of roles that they felt comfortable pursuing.

For P4, appearance was not seen as a barrier to employment. She shared, “You have a scar here? Oh, you can put more makeup there, right? Yeah, it’s not a problem. I think appearance is just outward; actually, there is no problem [with that]. It is your confidence that matters. And confidence level, it doesn’t come from how you look. It’s from inside. How you feel about yourself”. This perspective emphasises that self-perception and inner confidence can play a more crucial role than physical appearance in navigating professional environments.

P19 echoed a similar sentiment, attributing her positive employment experiences to a lack of emphasis on appearance by her employers. “I got this [current] job without interview. If I were unemployed and failed to get a job after many attempts, it would cross my mind [it is because of my appearance]. But because I didn’t go through that, so, I try not to think about it. And, I think, my employers, specifically, they never mention anything about it”. This absence of negative experiences allowed her to focus on her skills and performance rather than potential biases.

For P1, confidence and being “teachable” were viewed as more critical than appearance when seeking employment. “None of them asked me, eh, what happened to your lips? I have a scar, but none of them mentioned or brought up that issue. So, yeah, put on make-up and cover up a bit. Most importantly is that they want to see that you’re confident, and also I feel like one of the most important skills that you need to get the job is to be ‘teachable’.” Similarly, P6 expressed satisfaction with his academic achievement and current employment. He believed that completing his cleft-related treatments, such as lip and palate repairs and braces, contributed to his confidence. “I have a good job, and I am satisfied with it. I completed my lip repair and palate repair. Got my braces done. I have no issues”.

However, for some participants, societal biases around physical appearance played a subtle yet significant role in influencing the types of positions that they sought or the outcomes of their job applications. P15 reflected on the hospitality industry, where physical appearance is often prioritised, as follows: “They look at you, you have cleft. Then they feel you are not pretty, then you don’t get the job. Hotelier, mostly they look at your looks. So, they do consider your looks. I think had I asked to work at front office, I don’t think I would get the job”. P14 shared a similar experience when pursuing a job as an air stewardess. “I did try to get a job as an air stewardess. I passed a few rounds but in the last round I did not get the job because they did not like that I was wearing braces. I also felt they body-shamed me”. This reflects how rigid beauty standards in certain industries can disproportionately affect individuals with visible differences, such as cleft conditions.

These narratives highlight the complex interplay between personal confidence, societal perceptions, and professional opportunities. While many participants did not face direct discrimination, certain industries or roles that prioritise appearance posed challenges, leading some individuals to avoid public-facing positions.

#### 3.1.4. Sub-Theme 4: Health Concerns

Health issues associated with cleft lip and palate (CLP) can significantly influence job suitability and workplace safety, especially in environments that exacerbate physical vulnerabilities. The participants in this study shared how these health challenges affected their career choices and ability to sustain certain jobs, emphasising the importance of workplace accommodations and careful job selection.

P4 recounted the difficulties she faced as a diver due to incomplete cochlear development, a common issue linked to CLP. “Ever since I’ve been diving, [I had] no problem, except for ears. At that time, because my cochlea [development] is not complete. So, I have a tube inserted. So my problem is, if I dive, the water enters my ears and doesn’t come out like normal people. I am prone to get ear infection. That’s the only thing that’s actually quite hard for me. And my [diving] instructors are really worried about it. I still get ear infection now. Last few months, I actually ruptured my ear drums”. Her experience highlights how certain occupations, particularly those involving exposure to water or pressure changes, can pose serious risks for individuals with CLP-related health conditions. Despite her skills and interest in diving, these recurring infections and injuries created significant challenges for her safety and job sustainability.

Similarly, P17 shared how her work environment exacerbated her health issues, particularly before undergoing palate surgery. “At one time I was working with fabrics. I had particles coming into my nose. I was often sick and had the flu. At that time, I have not had my palate surgery. It was difficult for me to keep working there. I had to change jobs”. The inability to manage airborne irritants in her workplace led to frequent illness, ultimately forcing her to leave the job. Her experience emphasises the physical vulnerabilities that individuals with a CLP may face, particularly in roles that expose them to environmental irritants or other occupational hazards.

### 3.2. Theme 2: Psychosocial Factors

The researchers identified the following three psychosocial sub-themes impacting employment opportunities: traumatic childhood experiences, negative societal expectations, and low self-confidence. Despite being employed, many participants continued to face significant challenges related to these factors.

#### 3.2.1. Sub-Theme 1: Traumatic Childhood Experience

Childhood experiences significantly shaped the participants’ personalities and attitudes toward society, with many recounting difficult memories linked to their cleft condition. These early encounters often left lasting emotional scars, influencing their self-esteem and life choices. P15, for example, shared how persistent humiliation about her appearance caused deep embarrassment and self-consciousness, even around others with cleft. She explained, “Actually, I feel embarrassed to meet people with cleft. Like meeting all of you here now, very embarrassed. I don’t know why. Because, when we look at cleft person, ooh, do I actually look like that? I am not saying that person is ugly or what not. I mean, I am actually not pretty, right?” This sense of self-doubt, compounded by society’s emphasis on physical appearance, led her to avoid face-to-face roles, such as public relations or marketing.

For many, childhood teasing and bullying were universal experiences. P16 recalled, “The most difficult time was during primary school. Because kids are curious, asking why I’m like this, why their friend is like this”. However, as their peers matured in secondary school, these interactions became less common. P11 highlighted how speech difficulties made him a target for mockery, as follows: “When I was small, the children at my school would make fun of the way I speak. They would ask me to repeat, then they would mock me”. Similarly, P2, who also had a nasal deformity (rhinoplasty), reflected on the emotional toll of these encounters, as follows: “Back then, when we were kids, we cried a lot because every time we went out of the house, people would ask, why are you like that? Kids, right? Our circle of friends would say, eh, why is your nose like this? Why is my nose different? Why are you like this? And then they’d say, your teeth are crooked. Why do you speak unclearly and all that? So, I’d go home crying. I’d say, Mama, why am I different from everyone else? Why do people keep saying I’m ugly, mocking me like this? It really affected my social life because I didn’t want to go out of the house or mingle with anyone”.

Despite these adversities, the crucial role of supportive adults in raising resilience and confidence was also mentioned. P4 expressed gratitude for her mother’s efforts to prepare her for societal challenges, as follows: “As a cleft palate person, I’m very thankful to my mom, because since small, my mom really pushed me to be at the front…you need to show confidence when you go for storytelling”. Through activities like public speaking and storytelling, she built the confidence needed for job interviews and professional environments. Over time, these experiences nurtured a sense of self-assurance that helped to counter the negativity and self-doubt that had marked her childhood.

#### 3.2.2. Sub-Theme 2: Societal Expectation

Society’s perception of individuals with a cleft lip and palate (CLP) significantly impacts their confidence, self-image, and social interactions. A lack of public awareness about CLP often leads to misconceptions and uninformed judgments, which can create additional challenges for those with the condition. The participants in this study frequently shared how societal expectations shaped their experiences, particularly in relation to appearance and speech.

P15 emphasised the widespread lack of understanding about CLP in Malaysia, noting that many people remain unaware of the condition and its implications. “I think a lot of Malaysians don’t know cleft palate and cleft…what is it. So, what they don’t know, they don’t understand. So, those who are more aware of social issues, they won’t mind. But those who don’t, they—[find you] different. You look different or you don’t fit this and this”. This lack of awareness often leads to judgment based on superficial observations, with acceptance varying depending on educational exposure and environment. P15 remarked that people in more educated or urban areas and those exposed to online platforms tend to have a greater understanding and acceptance of visible differences, while individuals from less exposed areas may struggle to empathise.

For P2, the societal fixation on physical appearance was particularly painful during childhood, when hurtful comments about her looks led her to question her self-worth. “Once I started working, I can see the difference between those who are educated and the less educated ones on how they accept me [the way I am]. Those who are more higher educated are more accepting”. While adulthood brought about improved interactions in professional settings, she noted that misconceptions about her condition persisted. People often assumed that her appearance was the result of an accident or other external cause. “Whenever I’ve applied for jobs, no one has asked why my face looks like this. It’s only after starting work that people would ask, like, why? Is it because of an accident or did something else happen? So we just explain that it’s like this because we were born this way or due to operations or whatever”.

Speech challenges, a common issue for individuals with a CLP, further compounded societal expectations. P17 described how her nasal tone and difficulty pronouncing certain words before surgery affected her confidence. “Some words were hard to pronounce. So, when speaking, of course, it affects your confidence. And then, you’re afraid people won’t understand you or something. And then, when you have to do it in front of people, you feel like maybe they’ll think you’re not capable, because they cannot understand you”. These challenges not only influenced her self-esteem, but also her perceived ability to perform tasks in professional and social contexts.

#### 3.2.3. Sub-Theme 3: Lack of Self-Esteem

Self-esteem profoundly shapes the career trajectories of individuals with a cleft lip and palate (CLP), particularly when their physical appearance influences their confidence. Many participants in this study shared how insecurities about their appearance restricted their career options, limiting their potential and professional growth.

For P17, low confidence led her to avoid roles requiring public interaction. She recounted quitting her job at a hotel because she felt too self-conscious to perform in a visible position. “I was just behind the scene at that time. They asked me... they want to teach new kids [employees] to do the work. But at that time my confidence level was low. So I quit working there”. Her decision to remain in behind-the-scenes roles highlights the internal conflict between her professional aspirations and her insecurities. This hesitation to step into visible roles echoes a broader pattern of self-limiting choices driven by fear of judgment.

P15 shared similar concerns, explaining her reluctance to take on front-office positions due to societal beauty standards and the fear of being scrutinised. “People’s perception, some people are like this, some are like that. How perfect they want, we don’t know. But my group is a bit like that. Haaa, so they do stare. So, if I work at the front desk, I think I can’t”. This perception of being “different” and the anticipation of judgment create significant barriers, reinforcing a cycle of self-doubt and avoidance of roles that could offer growth opportunities.

P18 highlighted additional challenges related to self-esteem, stemming not only from her cleft condition, but also from financial constraints that prevented her from accessing surgeries like orthognathic procedures. “I work as a clerk. Sometimes I feel I can do better. Sometimes I think my cleft condition limits me, but I’m not sure. I may lack soft skills. I have problems with speaking clearly. I have not done my orthognathic surgery because I don’t have enough money”. Her narrative illustrates how unresolved physical concerns and speech challenges amplify feelings of inadequacy, further limiting her career aspirations.

### 3.3. Theme 2: Overcoming Challenges

The researchers identified the following two sub-themes that address the employment challenges that the participants faced: enhancing communication skills and boosting self-esteem. By focusing on these strategies, it was observed that the participants were able improve their ability to express themselves, overcome physical barriers, and build resilience, ultimately supporting their personal and professional growth.

#### 3.3.1. Sub-Theme 1: Improving Communication Skills

To address their speech and hearing challenges, the individuals adopted various methods tailored to their specific needs and circumstances. By adopting tailored strategies that addressed their unique needs, they could enhance their communication abilities and improve their overall quality of life.

For some, written communication served as a practical alternative to verbal interaction. P17 shared, “I prefer to write [when I want to communicate] rather than to speak. It’s like, ok, you don’t understand me, so I write it down for you”. This method not only ensures clarity, but also empowers people with cleft conditions to communicate effectively in situations where speech may fall short. Others relied on alternative forms of communication, such as sign language. P13 noted, “I learned sign language to communicate”, demonstrating the adaptability of individuals in bridging gaps in understanding. Hearing aids and lip reading also played a vital role in overcoming auditory challenges. P12 explained, “I can hear because I use [this hearing aid]. And sometimes I lip read too”. These tools helped the individuals to navigate environments where hearing impairments might otherwise limit their interactions.

Speech therapy and personal practices further contributed to improved communication. P14 reflected, “I go for speech therapy, and I realise sometimes my speech is not clear. To improve my speech, I will sing and record my singing. Then I will listen [to the recording] and learn where I need to improve”. This creative approach combines therapy with self-assessment, enabling individuals to build confidence and refine their speech.

Surgical interventions also had a transformative impact. P19 recounted her experience, stating, “After [palatal] surgery, like ok, my speech improved a bit, then easier to speak (and also eat). So, some things are like that. Actually, if we don’t do surgery, we can still survive, but if we do, our life improves. My friends say they can understand me better after I had the surgery”. For many, these procedures mark a significant milestone in their journey, providing tangible improvements in speech clarity and social interactions.

#### 3.3.2. Sub-Theme 2: Building Self-Esteem

The participants in this study shared insights on how they navigate their physical and psychosocial challenges and build self-esteem, demonstrating a journey towards positivity, undertaking self-reflection, and encouraging personal growth.

For some, self-affirmation played a critical role in maintaining a positive outlook. As P10 eloquently expressed, “We have to be positive. Convince ourselves that we can achieve better things in life. And we tell ourselves we are just like other people [who don’t have cleft]. And we can do what others can do too. We do a lot of self-talk. Positive talk. Pep talk”. This practice of reframing one’s inner dialogue provides a foundation for confidence-building strategies. The importance of surrounding oneself with supportive and uplifting individuals was highlighted by P5, who remarked, “Surround yourself with positive people. So that we don’t think about useless things. The main thing is God gives us sustenance so we must be positive about this”. This perspective stresses the value of a strong support network in fostering optimism and resilience.

The participants also shared how shifting their focus from personal struggles to broader perspectives helped them to overcome feelings of inadequacy. P14 reflected, “For me, when I’m feeling down, I think about how I don’t have [any problem] compared to other people who have lots of difficulties. Why should I feel down? Others have things worse than me, but they keep trying. I have [only] a little problem with cleft so I should keep on trying”. Personal stories also reflected a deep sense of pride in their skills and achievements, which further boosted their confidence. P7, a successful entrepreneur, shared, “I run my own business as a photographer and a designer. With my skill and experience, I build my own business. My customers are confident with my work. So just take care of yourself”. This narrative illustrates how developing expertise and earning the trust of others can empower individuals to thrive in their professional endeavours.

Physical improvements, too, played a role in enhancing self-esteem. P8, reflecting on her experience with braces treatment, noted, “I had the nasal sound before I started braces treatment. After that, people tell me I sound more clear when I speak. This makes me more confident to speak”. Such milestones not only improve speech intelligibility, but also contribute to greater self-assurance in social and professional settings.

## 4. Discussion

This study aimed to explore the employment challenges faced by young people with a cleft lip and palate (CLP), as understanding these obstacles is crucial for developing effective support and interventions. Related to this, an understanding of the participants’ background characteristics would add valuable insights into the self-identified employment challenges faced by individuals with a cleft lip and palate (CLP).

The predominance of women participants (17 out of 19) may reflect gendered differences in the willingness to share personal experiences in focus group settings. Men often adhere to traditional masculine norms that discourage the expression of vulnerability (Wagner & Reifegerste, 2024), which may explain their lesser representation in this study. This gender imbalance warrants further exploration, as the employment challenges faced by men with a CLP may differ due to societal expectations and gender roles. For example, the psychological consequences of workplace discrimination can differ between men and women. Women were demonstrated in another study to experience greater psychological distress due to the intersection of gender and appearance-based discrimination, affecting their perception of the condition’s impact on their overall well-being and job satisfaction (Tost et al., 2020).

An important finding was the relatively high level of educational attainment among the participants, with most having completed post-secondary education. This is consistent with studies indicating that individuals with a CLP can achieve significant academic milestones when supported by family, educators, and multidisciplinary care teams (Nicholls et al., 2018). However, this FGD finding may reflect selection bias, as individuals with higher education levels are more likely to participate in research studies (Kripalani et al., 2021). Furthermore, despite their educational achievements, most participants reported a monthly income from MYR 1000 to MYR 3000, which is below the national average for young professionals in Malaysia (Department of Statistics Malaysia, 2022). This finding aligns with previous research showing that individuals with a CLP often encounter barriers to career advancement, including stigma, discrimination, and limited access to professional networks (Havstam et al., 2011; Nicholls et al., 2018). It also raises concerns about the underutilisation of their skills and qualifications in the job market.

The diverse range of occupations and employment durations observed among the participants highlights the varied trajectories of individuals with a CLP. While some had successfully navigated employment challenges, others faced ongoing struggles, as evidenced by P10, a physiotherapy graduate seeking employment. These challenges may be linked to employers’ perceptions of physical appearance or speech differences, which can affect roles involving client interaction.

Through the thematic analysis of the participants’ narratives, this study reveals the complex interplay of physical, psychosocial, and societal factors that shape the employment experiences of young adults with a cleft lip and palate (CLP) in Malaysia.

### 4.1. Physical Factors and Employment

The physical challenges associated with CLP, such as speech intelligibility, hearing impairments, and health concerns, were significant determinants of employability among the participants. These findings align with the functional and communicative impairments among CLP individuals who do not receive comprehensive cleft care and are likely to grow up with speech and hearing disorders (Boonpiraks et al., 2024; Cheong et al., 2016).

The participants’ accounts revealed that speech, hearing, and appearance significantly influenced job opportunities, creating challenges frequently encountered by adults with a CLP (Stock et al., 2015). Speech difficulties, such as hypernasality and pronunciation issues, emerged as major obstacles, often leading to emotional distress and diminished confidence. Hearing impairments further contributed to communication challenges, sometimes requiring hearing aids or lip-reading. In certain sectors, appearance also impacted on the job search process, affecting the participants’ confidence and their willingness to seek customer-facing roles. CLP-related health issues, like recurrent ear infections, seem to be a recognised concern (Kurosaka et al., 2024). This observation warrants the need for careful job selection and workplace accommodations to address these physical concerns.

### 4.2. Psychosocial Factors

The psychosocial impact of CLP, including childhood trauma, societal expectations, and self-esteem, was another significant theme. The participants’ accounts of bullying and social exclusion during childhood, such as P15’s experiences, resonate with findings from the literature, which highlight that societal perceptions and stigma surrounding facial differences can adversely impact the social interactions and self-image of affected individuals (Salinero et al., 2024). Common stigmatising themes, including labelling, where children were mocked for physical traits such as speech and facial differences, were consistent with those reported in a Colombian study, and this could lead to the avoidance of social interactions (Arias-Urueña et al., 2023). Such verbal abuse may profoundly influence self-perception and, subsequently, career choices, often leading individuals to avoid roles involving extensive face-to-face interaction (Nicholls et al., 2019b). Similar experiences and societal stigma surrounding physical differences are universal challenges faced by individuals with visible differences, suggesting the necessity for global awareness and intervention strategies.

Societal expectations about appearance further shaped the participants’ career trajectories, particularly in industries that prioritise aesthetics. P15 and P14’s experiences with biases in the hospitality and aviation sectors align with Western research demonstrating how beauty standards disproportionately disadvantage individuals with visible differences (Hosoda et al., 2003). However, the findings also highlight that participants like P19 valued confidence and skills over appearance in less appearance-focused jobs, showing that building resilience and self-confidence can help to counter societal biases.

Low self-esteem, frequently rooted in early life experiences, emerged as a critical barrier to employment. Participants like P17 and P18 described avoiding public-facing roles due to fear of judgment, reflecting the findings by Rumsey and Harcourt (Rumsey & Harcourt, 2004), who stressed the impact of self-perceived body image on professional success. However, the participants’ emphasis on the role of family and community support in building confidence adds a culturally specific dimension, aligning with Asian collectivist values that emphasise the importance of familial and social networks in shaping individual outcomes (Gifalli et al., 2024).

### 4.3. Strategies for Overcoming Barriers

This study further explored how participants with a CLP overcame employment obstacles by enhancing their communication skills and self-confidence. The participants adopted various strategies to address speech and hearing challenges, including written communication, sign language, hearing aids, speech therapy, and, for some, surgical interventions. These approaches helped them to navigate communication barriers and boost their confidence in professional settings. Self-esteem emerged as a crucial factor in managing both physical and psychosocial barriers. The participants used positive affirmations, relied on supportive social networks, and practiced self-reflection to build resilience, which Raptis (Raptis, 2023) considered as trauma-informed practices to build resilience.

Fennell’s Cognitive Theory of Self-Esteem suggests that foundational beliefs about oneself are shaped by early experiences (Fennell, 1997). Negative experiences often foster limiting beliefs, while positive ones build confidence and resilience. Applying Fennell’s theory, these limiting beliefs may be overturned through communication training (e.g., speech therapy) and assistive technologies (e.g., hearing aids), which serve to build self-esteem by enhancing individuals’ communication skills and reducing their social anxiety. Self-esteem development programs, such as workshops and mentorships, can play a crucial role in promoting resilience and fostering a positive self-image. Additionally, inclusive workplace policies are essential for sustaining a positive self-esteem and support among individuals with a CLP. Public awareness campaigns aimed at reducing stigma around CLP could further promote societal understanding and acceptance, fostering a more inclusive employment landscape (Kalra, 2014).

The self-esteem-building strategies identified in this study also exhibited both universal and context-specific elements. While practices like positive self-talk and surrounding oneself with supportive individuals, as described by P10 and P5, align with Western psychological approaches (Searle et al., 2017), the participants also emphasised the role of cultural and religious values in fostering resilience. For instance, P5’s focus on divine providence as a source of positivity reflects the influence of spirituality in shaping coping strategies in Malaysia, a dimension less emphasised in the Western literature.

### 4.4. Policy and Practical Implications

These findings highlight the need for workplace policies that accommodate the specific needs of individuals with a CLP. Employers should consider implementing training programs for staff to promote awareness and understanding of CLP, creating a more supportive environment. Additionally, inclusive work-based practices, such as a supportive culture, job accommodations, and performance management, positively influence the employment and retention of vulnerable workers, including those with unique challenges—this is supported by a recent scoping review on organisational policies related to the labour force (Kersten et al., 2023). By promoting awareness and understanding, these policies can reduce biases and improve access to employment opportunities. On a separate note, it is desirable for healthcare systems to integrate or link career counselling and support services for individuals with a CLP, ensuring that they have access to the resources necessary for successful employment. Effective strategies to build self-esteem for adults with a cleft lip and cleft palate include receiving sufficient explanations about their condition from parents and healthcare providers, as outlined by the works of Omiya et al. (Omiya et al., 2012).

### 4.5. Limitations and Future Research

This research offers valuable insights into the employment challenges identified and defined by young adults with a CLP, but it has certain limitations. The predominance of female participants and those with post-secondary education introduces an imbalance that may affect the generalisability of the findings. Additionally, the study did not examine labour market policies or practices, which are crucial for understanding systemic barriers. Future research should address these gaps by including larger, more diverse samples across gender, education levels, and cultural contexts to better capture the broader implications of these challenges. Furthermore, exploring the labour market’s demand side—such as employer attitudes, hiring practices, and workplace inclusivity—could provide a more comprehensive understanding of how societal biases and structural factors shape employment opportunities for individuals with a CLP.

## 5. Conclusions

This study highlights the multifaceted employment challenges faced by individuals with a CLP, encompassing speech, hearing, appearance, and psychosocial factors, which impact career opportunities and professional integration. Addressing these barriers requires a comprehensive approach focused on enhancing communication skills and building self-esteem. Key recommendations include targeted interventions such as specialised career counselling, the provision of assistive technologies, and the implementation of inclusive workplace policies to foster greater accessibility and support. Increasing public awareness is also essential in combating societal stigma, as it helps to cultivate a more understanding and supportive environment for individuals with a CLP. Such awareness initiatives, combined with targeted policies, can promote equity and inclusivity, enabling individuals with a CLP to thrive in their professional lives and contribute meaningfully to society. These measures collectively represent a proactive strategy for addressing CLP-related employment challenges and advancing a culture of inclusivity and opportunity.

## Figures and Tables

**Table 1:** Background of FGD participants.

No	Gender	Age	Monthly Income	Education	Occupation	Years in Current Employment
P1	Female	25	MYR 1001–3000	Degree	Corporate communications executive	1 year
P2	Female	25	MYR 1001–3000	Degree	Airport apprentice	4 months
P3	Female	28	MYR 1001–3000	Secondary school	Factory operator	7 months
P4	Female	28	>MYR 3000	Degree	Diving instructor	9 years
P5	Female	19	Not applicable	Ongoing diploma	Student	Not applicable
P6	Male	26	MYR 1001–3000	Degree	Welding engineer	2 years
P7	Female	29	>MYR 3000	Diploma	Business owner	4 years
P8	Female	31	>MYR 3000	Diploma	Government administrative	4 years
P9	Female	33	MYR 1001–3000	Degree	Science officer	10 years
P10	Male	23	Not applicable	Degree	Unemployed	Not applicable
P11	Male	22	MYR 1001–3000	Secondary school	Factory machine operator	6 months
P12	Female	24	MYR 1001–3000	Secondary school	Supermarket cashier	3 years
P13	Female	27	<MYR 1001	Secondary school	Children’s nursery caregiver	1.5 years
P14	Female	28	<MYR 1001	Diploma	Clerk	4 years
P15	Female	28	>MYR 3000	Degree	Hotelier	8 years
P16	Female	38	>MYR 3000	Degree	Administrative executive	10 years
P17	Female	32	MYR 1001–3000	Diploma	Business owner	1 year
P18	Female	21	MYR 1001–3000	Diploma	Clerk	1 year 3 months
P19	Female	26	MYR 1001–3000	Degree	Social media executive	1 year 6 months

**Table 2:** Themes and Sub-themes.

Themes	Sub-Themes
Physical factors	Speech intelligibility
	Hearing impairment
	Perceptions of appearance
	Health concerns
Psychosocial factors	Childhood experiences
	Societal expectations
	Lack of self-esteem
Overcoming challenges	Enhancing communication skills
	Building self-esteem

## Data Availability

Data supporting reported results shall be made available upon reasonable request.
